# Prescription and Integration of Accredited Mobile Apps in Catalan Health and Social Care: Protocol for the AppSalut Site Design

**DOI:** 10.2196/11414

**Published:** 2018-12-21

**Authors:** Francesc López Seguí, Carme Pratdepàdua Bufill, Ariadna Rius Soler, Marc de San Pedro, Bernat López Truño, Agnès Aguiló Laine, Jordi Martínez Roldán, Francesc García Cuyàs

**Affiliations:** 1 TIC Salut Social Generalitat de Catalunya Mataró Spain; 2 Centre for Research in Health and Economics Department of Experimental and Health Sciences Universitat Pompeu Fabra Barcelona Spain; 3 Universitat de Vic – Universitat Central de Catalunya Vic Spain; 4 IN2 Barcelona Spain

**Keywords:** mHealth, information integration, telemedicine, telemonitoring, mobile phone

## Abstract

**Background:**

The use of new mobile technologies in the health and social welfare sectors is already a reality. The ICT Social Health Foundation, in accordance with the technology strategy of the Catalan government’s Ministry of Health and its Ministry of Labour, Social Affairs and Families, is leading an initiative to create a public library of apps for its AppSalut Site.

**Objective:**

The objective of this paper is to present an account of the design of the project, with a global perspective, applied to the Catalan ecosystem, which can be divided into 3 areas: the framework governing the recommendation and prescription of apps, the subset of interoperability for mobile environments, and the data storage infrastructure.

**Methods:**

The security and credibility of the apps included in the catalog is ensured by submitting them to an accreditation process in the public domain that provides users with the guarantee that they are fit for purpose and trustworthy for the management and care of their health, while providing health care professionals with the possibility of recommending the apps in the doctor’s surgery, as well as adding the information generated by the users’ mobile devices to the information systems of the various organizations concerned.

**Results:**

An examination of the abovementioned areas suggests possibilities for improvements in the future. The experience obtained from the development of this element has shown the heterogeneity of the vocabularies used, as expected, due to the lack of awareness on the part of the developers regarding the need to standardize the information generated by the app, requiring the foundation to take on the role of consultant.

**Conclusions:**

The project has evolved in keeping with changes in the technological and social paradigm and responds very satisfactorily to the needs posed to it. It can be seen as a landmark experience in mobile strategies in the fields of health and welfare of any public health system. The experience has shown itself to be feasible in organizational terms, necessary in any attempt to integrate mobile technologies into public health practice, and a global pioneer in the field.

**International Registered Report Identifier (IRRID):**

RR1-10.2196/11414

## Introduction

### Background

An increase in the use of new mobile technologies in the field of health care has been thoroughly documented. *The Green paper on mobile Health* [1] describes mobile health (mHealth) as “an emerging and rapidly developing field which has the potential to play a part in the transformation of health care and increase its quality and efficiency.” More recently, the *mHealth App Economics* report [2] predicted that in 2021, health apps would be recommended by health care professionals by means of specialized portals as part of routine visits to a doctor. The 2017 edition of the report [3] estimates that 325,000 mHealth apps are available to download from official stores (AppStore, Google Play, etc). The *A Connected Digital Single Market* [4] report points to the increasing ability of patients to upload their medical records to their electronic medical history and to interact with health care providers.

Despite a wealth of evidence regarding the degree of implementation of mHealth and its potential, few public initiatives are promoting specific strategies in the field of health app libraries; at the national level, the United Kingdom’s National Health Service has its Digital Apps Library, which is still at the design stage, while at the regional level, the Andalusian Health Service maintains its *catalog of mobile health apps*, also standing for an accreditation process [5]. Catalonia, a pioneer in the adoption of mHealth policies, recently approved its Strategic Mobility Plan [6], urging the institutions involved in the sector to bring health care and social welfare services to the public via mobile technologies and to facilitate the transformation of the various social and health care processes to achieve integrated attention, which more effectively meets patients’ needs. In response to this objective, the ICT Social Health Foundation created an apps library for the health and social care sectors called AppSalut. The foundation certifies that the apps featured in its library are suited for facilitating and improving the monitoring of patients’ health. This guarantee is achieved by submitting all the apps to an accreditation process created by the foundation , which is in the public domain; the apps are subsequently made available to doctors who can recommend them during a consultation in order that the information generated can be added to the institutional information systems as an aid to making decisions. Furthermore, the AppSalut Site was created to help empower patients, making them aware of and responsible for their own health, while ensuring that information moves in both directions, between patients and their health care team.

This paper outlines the comprehensive, forward-looking solution developed by the ICT Social Health Foundation. It details the design of the project and the 3 elements on which it is based: the framework governing the recommendation and prescription of apps, the subset of interoperability for mobile environments, and the data storage infrastructure. This paper concludes with how the project will develop and possible changes in the future.

### Objectives

The aforementioned speed with which mobile technologies have been adopted in the fields of health and social welfare implies a series of needs that the AppSalut Site has defined in terms of challenges, which are shown in Table 1.

**Table 1:** Needs and challenges in relation to the AppSalut Site.

Needs	Challenges
The app stores feature an enormous number of apps that are highly heterogeneous in terms of quality and type. A trusted framework is required to ensure that both doctors and patients use apps that meet certain minimum requirements.	Certify the app’s relevance and added value by means of an accreditation process.
The apps also capture and record data by means of associated devices (glucometers being the most frequent).	Standardize the devices linked to mobile apps.
Every device uses its own platform, meaning that doctors who wish to access the data generated by their patients must enter the same number of platforms as devices used by their users.	Design a single platform for viewing all the collected data and adding it to the health system’s information system.
The aforementioned apps and devices collect data without necessarily adhering to international standards.	Standardize the data from the apps through the use of an interoperability framework.
The collected data cover very different themes (related to lifestyle, physical activity, etc, both actively and passively) and its veracity must be proven.	Upload data solely at the doctor’s discretion and distinguish it visually from the rest of the patient’s medical history.
From the start, developers should become aware of the desirability and potential added value of incorporating information from the app with public information systems and that they take this into account during the app’s technical design.	Support developers in the design of apps that are likely to be added to the library.
The prescription of mobile apps in the health environment is currently carried out on an informal basis: the doctors prescribe those they know or have been recommended to them by other professionals.	Create an institutional prescription process and integrate the recommendation of apps into clinical information systems.
Apps can serve to empower citizens in taking care of their health, and they can generate quality doctor-patient communication, giving a much more holistic vision of health, oriented toward promotion and prevention.	Organize the process around the Personal Health Folder.

In relation to the aforementioned challenges, and for the purpose of simplifying this account, 3 basic lines of action have been established, in the form of objectives: (1) the creation of a Web interface that outlines the process of accreditation and the prescription of apps, which serves as a point of contact with the app developers; (2) the creation of a framework of mHealth standards and interoperability; and (3) the implementation of a structured data repository that can be consulted both by patients and doctors and services that allow the stored information to be exploited. These objectives are specifically addressed through the structural elements that collectively make up a new health care process, which is made available to patients, known as the AppSalut Site.

## Methods

### The Framework for Interaction With Developers, Professionals, and Users

The first 2 processes that need to be systematized require an element of interaction between the team in charge of the project, the apps’ developers, and the professionals who need to use them. In the first instance, the accreditation of the apps (the certification of their suitability for use) involves a process in which they must meet a series of conditions (not solely of a technological nature). In the second instance, a process needs to be designed through which professionals can recommend (or “prescribe”) these certified apps to their patients (detailed below).

#### Accreditation Process

The design of the accreditation process must be based on a consensus between professionals in the sector: experts in the field of technology, health professionals (doctors, nurses, psychologists, and social workers), health communication professionals, expert patients, institutional representatives, and members of the public. This multidisciplinary approach ought to be shaped by working groups and meetings, with two major objectives: reaching a consensus on the stages of the accreditation process and the creation of a list of evaluation criteria. With reference to the first question, 5 different stages are to be taken into consideration:

1. *New apps*: consideration of a new app for approval by means of a preliminary form, filled out by the developer, containing certain basic information
2. *Initial validation*: meeting of certain preliminary requirements (eg, if the app is operational and whether it works correctly)
3. *Classification*: categorization according to the app’s complexity (which may determine the type of evaluation that needs to be carried out)
4. *Evaluation*: a request is made to a committee of experts for them to apply a series of criteria, corresponding to different areas, by which they can evaluate the app
5. *Result*: if the procedure is satisfactory, the corresponding certificate of accreditation is generated and published in the app library

At the end of the process, the app is awarded a numerical score that is published in the AppSalut Site. The accreditation remains valid as long as the app is active or until changes are made to its content and functionality; consequently, those responsible for the app will have to commit to reviewing the process if such changes occur.

#### Recommendation Process

With regard to the recommendation process, 2 considerations must be met. First, it must be remembered that the prescription of the app must be properly integrated, both occurring as part of the currently existing patient clinical pathway and forming part of the information system used by clinicians. Second, it must be emphasized that mHealth technologies have largely focused on the management of chronic diseases, where continuity becomes a fundamental aspect [7]. It seems reasonable, therefore, that the starting point for these apps is during a consultation with the primary health care physician, where there is greater potential for the attributes of telemonitoring and the observation of chronic patients.

### Data Interoperability

Interoperability is the ability to share information between components (such as systems or devices) without its meaning being lost. This communication must ensure the coherent exchange of data among departments, organizations, health care levels, or regions. Its main objective is to provide doctors with relevant information regarding their patients to ensure that the decision-making process takes place in a safe, efficient, and effective manner. Interoperability guarantees access to information regardless of where it was recorded, thus, favoring its reuse, minimizing blind spots, and ensuring the continuity in health care. Standard health interoperability is widely used and studied [8], although it is still emerging in the mHealth field. This is the second of the 3 elements that need to be developed in the framework of the AppSalut Site.

The chapter devoted to mHealth in the study entitled *From Innovation to Implementation, eHealth in the WHO European Region* [9] shows that more than half of the health centers that were surveyed promote the use of standards and interoperability in mobility, demonstrating that they form part of the health systems’ agenda. A global pioneer, the Catalan model of interoperability in mHealth [10,11], focuses mainly on layers of syntactic interoperability (relative to the structure and format of the information to be exchanged) and semantics (which guarantees that the information maintains its integrity at the receiving component). With regard to the first of these levels, in traditional models of information exchange, Health Level 7 (HL7) messaging is commonly used for the notification of information and occasional events, such as in *Clinical Document Architecture-Release 2* documents for the sharing of clinical reports. Both standards are based on the exchange of XML files, and although they are specific to the health sector, they are not suitable for mobile apps. In contrast, the Fast Healthcare Interoperability Resources (FHIR) standard, also by HL7, was created to respond to new information-sharing needs, allowing the use of Representational State Transfer (REST) Web services in *JavaScript Object Notation* format. This standard allows the sending of only the relevant and/or necessary information, simplifying the exchange of health information and adapting the methods of transmission to the mHealth environment.

In terms of semantic interoperability, apart from sharing information between the apps and the central data repository, it is also essential that the data exchanged can be compared and exploited globally. As a result, it is imperative that if two apps register the same data, such as weight, they do so based on the standardized criteria that define the code, the type of data involved, and what units of measurement it represents. Based on a current state review of all the available controlled vocabularies, Systematized Nomenclature of Medicine–Clinical Terms (SNOMED-CT) has been chosen as a reference terminology due to its flexibility and enormous scope.

### Data Repository

The data repository, as the third and final element that should make up this new care process, must store information from mobile devices for later use, either by doctors as part of health and social care systems and/or by patients themselves. Therefore, this requires a platform that can address the 3 Vs common to solutions based on Big Data: volume (a large number of mobile apps, users, and data are generated), velocity (data are captured in real time), and variety (every device structures information in a unique, heterogeneous manner, so the way in which it is stored must adapt to this diversity of origins).

To the aforementioned “Vs,” we need to add two specific ones that are of great relevance in the health and social welfare sectors: veracity (it is necessary to ensure that the stored data come from a reliable source of information) and value. The information stored on the platform must allow the following uses: (1) for doctors, to personalize the treatment for patients, with the use of information originating from personal devices; (2) for patients, to visualize the information from mobile apps they have installed in a standard manner; (3) for the current information systems, to add the necessary information to patients’ clinical history; and (4) for the remaining agents in the sector, the exploitation of the data, creation of alerts, prevention, and prediction.

## Results

### The AppSalut Site

In terms of the structure outlined in the previous section, the following is the outcome proposed by the ICT Social Health Foundation, in accordance with the specific characteristics of the Catalan public system. The process of defining the project has transformed the 3 identified needs into 3 corresponding products designed for the Catalan context and adaptable to other health systems.

In relation to the framework for interaction with developers, doctors, and users, the AppSalut Site [12] was created; it is a Web interface acting as an *mHealth Hub* for clinicians; app developers; patients; and the sector, where guidelines, rules of use, and news are published. The AppSalut Site backstage is, simultaneously, the ICT Social Health Foundation’s management tool. Below is a summary of how the accreditation and recommendation processes are organized around this Web portal.

#### App Accreditation

The expert consensus on the app accreditation consists of 2 processes: first, a task force is dedicated to specifying the means of classifying the apps, based on the foundation’s participation in the *CNECT mHealth Expert* groups and the mHealth subgroup of the European Commission’s *eHealth Network*. Three parameters have been defined for evaluation, namely, the impact of use (in terms of the volume of users potentially susceptible to using the app in the Catalan context), the type of health recommendation that the app performs for users (more or less specific according to the use of the data supplied by users), and the type of sensitive information that is registered (a critical aspect to take into account because certain apps do not transmit data). These 3 indicators determine the requirements of the accreditation process, establishing which of the recommended or desirable criteria are obligatory, according to the 3 levels, whereby the obligatory criteria are essential for the app to pass the accreditation process (more detailed information on this aspect is provided in the Multimedia Appendix 1).

Second, a total of 5 working sessions with the expert groups and the advice of a specialized technology consultancy have resulted in the publication of 120 evaluation criteria that are grouped into 4 areas. First, functionality: the evaluation of the quality and utility of the app’s contents. These are made up of 25 criteria and are given priority over the following 3 areas. Second, usability and design: accessibility, user experience, and visual aesthetics. Third, technology: technological reliability and adaptability of the app in general. Last, security: guarantee of data security and adherence to data management policies. The experience to date indicates that the technology and security areas usually work well, while functionality and usability are more critical (specifically, the latter shows that most of the analyzed apps had shortcomings in terms of accessibility for users with functional diversity). In light of these criteria, the evaluations are conducted by two committees, whose structure is summarized in Table 2.

In addition, those responsible for the evaluations are charged with developing proposals for modifying or improving the criteria. It is desirable that developers consider them in early stages of the design of apps, as far as possible, as their usability may be affected by being adapted to meet the requirements (eg, including a specific log-in process). With the publication of the criteria, guides, recommendations, and seminars aimed at providing support for developers, and help from all the relevant entities involved in the sector, the foundation has opted for an open, transparent accreditation process, which prioritizes the gaining of trust from patients and doctors.

**Table 2:** Constitution of the app accreditation committees.

Committee	Members
Functional (in charge of the functional criteria)	Official College of Doctors of Barcelona Official College of Nurses of Barcelona Catalan Society of Clinical Psychologists Association of Family and Community Nursing Catalan Society of Family and Community Medicine Official Association of Graduates in Physical Education and Physical Activity and Sports Sciences of Catalonia
Technical (in charge of the usability and design, technology, and security criteria)	Currently managed by a specialized consulting company

#### Recommendation

The recommendation process is the route by which doctors can prescribe apps to users. As mentioned above, primary health care has been identified as the most appropriate process for monitoring the information recorded, with the general practitioner being identified as the principal agent responsible for the prescription process. In addition, it is the most technologically feasible option, given the needs of the specific case in question, as >90% of Catalan Primary Care Teams use the same clinical management software (named *eCAP*). The prescription process involves the stages identified in Textbox 1.

### The Digital Health Platform

The Digital Health Platform (DHP) has been designed and implemented as a data repository that allows doctors to access all the information generated by the apps at any time and check it, if applicable, before it is included in the information system. It is primarily based on solving the stated objectives in a modular, interoperable way. The following are the main areas: (1) data storage, which includes the apps that have been recommended to patients, the catalog of variables, and the data originating from the mobile apps; (2) services for interacting with the data storage; and (3) the corresponding identification services. The following sections concern the sequence of tools presented in Table 3.

With regard to the access to information stored in the DHP, the system’s architecture provides the possibility of allowing third parties to access the DHP’s information system to upload data to their own repositories, but this has yet to be implemented.

### The Software Development Kit and the Mobile Subset for Interconnecting With a Common Vocabulary

Based on the identified information exchange needs, the following interoperability devices have been developed. First, a Software Development Kit that will evolve into an FHIR implementation (this standard will be gradually phased in). Initially, a much simplified first version was created to send variables of the “identifying-value” kind. Its technical aspect was tested at a hackathon specifically intended for this purpose, allowing developers to assess the feasibility of uploading data from 14 different apps to the DHP, in a controlled environment [13].

The second defined interoperability device focuses on the semantic layer and consists of a subset of mHealth and mSocial mobility variables. The three main challenges presented by mobile technology are the great diversity data types, the rapid responsiveness required, and the app developers’ lack of familiarity with the controlled vocabularies. As a result, the methodology used to standardize the controlled vocabularies also needs to be modified; to add an app to the AppSalut Site, its variables are analyzed and standardized in a specific way, assigning it a set of codes relating to the data types and the corresponding units of measurement. To help developers identify and standardize which variables to send, a notification template of variables has been created and information sessions have been held to emphasize the importance of applying the standards. As a result of these efforts, a subset of mHealth and mSocial mobility variables of SNOMED-CT has been defined, which has >50 concepts originating from 6 apps. This subgroup ensures that data exchanged between the apps and the DHP can be compared and exploited globally. Other vocabularies such as Logical Observation Identifiers Names and Codes, International Classification of Diseases-release 10, and Anatomical Therapeutic Chemical Classification are also taken into account, but these are mapped to SNOMED-CT to have a common base of reference terminology. The subset should, therefore, be seen as a dynamic tool that requires a versatile standard (FHIR).

The experience obtained from the development of this element has shown the heterogeneity of the vocabularies used, as expected, due to the lack of awareness on the part of the developers regarding the need to standardize the information generated by the app, requiring the foundation to take on the role of consultant.

**The prescription stages in the AppSalut design.**

**Recommendation**

During the first visit, the health professional recommends a mobile app to a patient. To do so, the doctor accesses eCAP and once logged on, prescribes an app available on the AppSalut Site without the need to log on a second time. The patient then receives a short message service (SMS) text message on his or her mobile phone containing a registration code, allowing him or her to identify himself or herself when sending data.

**Download**

The user can download the app directly from official app stores (Google Play or AppStore) or through his or her Personal Health Record “My Health” account, where the prescription will have been registered.

**Registration**

Once the app is installed on patients’ mobile device, they need to enter the code they were sent via SMS text message and accept the conditions of the service, thus, agreeing to have their data stored on a public network.

**Setup**

Depending on which app has been recommended, patients can activate a series of alerts on their device to warn them, if necessary, when they need to enter their information. The specifics of this stage depend on the app in question.

**Uploading data**

Apps automatically upload the information on a continual basis. Both patients and doctors can access their respective viewers in the AppSalut Site, which displays information relating to all the apps linked to AppSalut in the form of graphs and allows them to apply filters (date, time, etc).

**Evaluation of the results**

Doctors can visualize the information collected while patients have been using the app and select the data to be included in their clinical history.

**Table 3:** Tools and needs of the Digital Health Platform.

Tools	Needs
Nonstructured Query Language-type database	To store large volumes of unstructured data without having to predefine its structure
Representational State Transfer (REST) services	To allow mobile apps to upload data to the database
Credential validation services	To request the identification services to confirm the identity of a patient or a doctor
Simple Object Access Protocol and REST services	To allow users to interact with the stored data, either checking it or consulting it
Encryption and decryption service based on a public or private asymmetrical key	To ensure the veracity of the origin of the data
Software Development Kit	To allow app developers to access all of the platform’s features concerning the inclusion of their data
A message in a custom format based on Health Level 7	To allow data from the apps to be added to the repository. The current message always has the “Variable-Value” structure where the variable name is its code in the corresponding catalog (Systematized Nomenclature of Medicine–Clinical Terms; the International Classification of Diseases, Ninth Revision, etc). The Software Development Kit sends the date and time of when the specific value of the variable has been registered
An identity server	To facilitate the creation and configuration of all of these services, both in the validation of credentials or tokens, as well as in the delegation and federation of the credentials

## Discussion

This experience illustrates that an accreditation model is a key structural element of the prescription and data integration process. It must be conceived in a highly dynamic fashion, open to feedback from the agents concerned and adaptable to technological changes, plus the concerns of both app developers and patients. The multiplicity of actors related to social and health care assistance and the recent increase in devices connected to apps highlight the need for the process described to be dynamic and modular, in spite of its complexity. The certification of glucometers, for example, has become an important practice that has led to new challenges. In the same way, due to the interoperability framework, the proposed solution is an excellent starting point for a path that is gradually becoming defined; it is necessary to both expand the commonly controlled vocabulary in mobile technology (allowing developers to code according to international standards) and guarantee a communication protocol for mobile technologies regulated by an official body to face the contradiction of building local interoperability solutions to global products. The project’s feasibility was recently confirmed in a pilot study lasting 5 months, conducted with 4 Primary Care Teams belonging to the Catalan public health system [14]. Further investigation needs to be addressed to record these first-stage experiences in the mHealth public prescription field.

The results obtained demonstrate the success of the AppSalut Site; the project has evolved in keeping with changes in the technological and social paradigm and responds very satisfactorily to the needs posed by the analysis undertaken by the Catalan government’s Ministry of Health and its Ministry of Labour, Social Affairs and Families in their Strategic Mobility Plan. It can be seen as a landmark experience in mobile strategies in the fields of health and welfare of any public health system. The experience has shown itself to be feasible in organizational terms, necessary in any attempt to integrate mobile technologies into public health practice, and a global pioneer in the field.

## Appendix

Multimedia Appendix 1 App classification, by level.

## Abbreviations

DHP: Digital Health Platform
FHIR: Fast Healthcare Interoperability Resources
HL7: Health Level 7
mHealth: mobile health
REST: Representational State Transfer
SMS: short message service
SNOMED-CT: Systematized Nomenclature of Medicine–Clinical Terms

## References

[1] (2014). European Commission.

[2] (2016). Research 2 Guidance.

[3] (2017). Research 2 Guidance.

[4] CalidadAppSalud.

[5] (2017). European Commission.

[6] mHealth.cat Mobile Master Plan Strategy and Action Plan: Improving public healthcare and social well-being services.

[7] Hamine S, Gerth-Guyette Emily, Faulx Dunia, Green Beverly B, Ginsburg Amy Sarah (2015). Impact of mHealth chronic disease management on treatment adherence and patient outcomes: a systematic review. J Med Internet Res.

[8] Benson T (2010). Principles of health interoperability HL7 and SNOMED.

[9] World Health Organization (2016). From Innovation to implementation: eHealth in the WHO European Region.

[10] Rius A, Pratdepàdua C (2015). Diccionario Clínico para iSalut. Papeles Médicos, vol.23 num.2.

[11] Rius A (2012). Una Experiencia de Creación de Subconjuntos en SNOMED CT.

[12] SmartCatalonia (2017). Mediktor and Meplan earn the first mConnectathon in ICT, Health and Social.

[13] SmartCatalonia AppSalut.

[14] Lopez Segui Francesc, Pratdepadua Bufill Carme, Abdon Gimenez Nuria, Martinez Roldan Jordi, Garcia Cuyas Francesc (2018). The Prescription of Mobile Apps by Primary Care Teams: A Pilot Project in Catalonia. JMIR Mhealth Uhealth.

