# Supplementary material for: Prescription and Integration of Accredited Mobile Apps in Catalan Health and Social Care: Protocol for the AppSalut Site Design
Source: JMIR Res Protoc. 2018 Dec 21;7(12):e11414. doi: 10.2196/11414 (PMC6320420; doi:10.2196/11414)
Supplement: Multimedia Appendix 1 [file resprot_v7i12e11414_app1.pdf]

Appendix 1: Classification by level

In order to determine which criteria apply in the accreditation of an app, two general aspects (technological and content) have been evaluated, which in turn are analysed according to the risk of three factors: the sensitivity of the information being managed, the type of recommendation being made and its impact.

A. Sensitive Information: apps can manage private information that could jeopardize patient's privacy. This factor classifies apps according to how information is dealt with:

- Low: the app does not collect data
- Moderate: the information is kept locally, or the collected data is transmitted in aggregate form
- High: the application collects user data that is transmitted outside the app

B. Health information or recommendations: the information provided by the app may be incorrect. This aspect classifies them according to the potential risk of this information:

- Low: the app does not provide information about the patient's health status, makes no recommendations or provides specific information about the current state of the user or their activity
- Moderate: the app provides information but it is decontextualized, it is general information or originates from standard protocols and recommendations
- High: the app provides information or specific recommendations for the user based on the data collected by the application or entered by the user

C. Impact: this factor classifies the app according to the relative risk to the potential number of users of the app (assuming the Catalan population is approximately 7,5 million):

- Low: potential users less than 2% of the population (less than 150,000)
- Moderate: potential users between 2% and 10% of the population (between 150,000 and 750,000)
- High: potential users exceed 10% of the population (over 750,000)

The categorization of these factors results in the classification of each of the aforementioned areas into three levels, as shown in the following table:

	Impact				
	Low	Moderate	High		
**Technological aspect**	1	1	2	Low	*Sensitive information*
	2	3	2	Moderate	
	3	3	3*	High	
**Content aspect**	1	1	2	Low	*Information or health recommendations*
	1	2	2	Moderate	
	2	3	3	High	

*Table 2. Level of demand for technological and content aspects as a combination of impact, sensitive information and health information or recommendations factors. *In this particular instance, the application of the criteria also contains a specific layer of security.*
